# Assessment of Lead (Pb) Toxicity in Juvenile Nile Tilapia, *Oreochromis niloticus*—Growth, Behaviour, Erythrocytes Abnormalities, and Histological Alterations in Vital Organs

**DOI:** 10.3390/toxics10120793

**Published:** 2022-12-16

**Authors:** Tayeeba Ferdous Mahi, Gourab Chowdhury, Mohammad Amzad Hossain, Asim Kumar Baishnab, Petra Schneider, Mohammed Mahbub Iqbal

**Affiliations:** 1 Department of Fish Biology and Genetics, Sylhet Agricultural University, Sylhet 3100, Bangladesh; 2 Department for Water, Environment, Civil Engineering and Safety, Magdeburg-Stendal University of Applied Sciences, Breitscheidstraße 2, D-39114 Magdeburg, Germany

**Keywords:** Pb toxicity, HSI, erythrocytes, histopathology, *Oreochromis niloticus*

## Abstract

Lead (Pb) is one of the toxins responsible for the deterioration of ecological health in aquatic environments. The present study investigated the effects of Pb(NO_3_)_2_ toxicity on growth, blood cell morphology, and the histopathology of gills, liver, and intestine of juvenile Nile tilapia, *Oreochromis niloticus*. A 30-day long aquarium trial was conducted by assigning three treatment groups T_1_ 5.20 mg L^−1^, T_2_ 10.40 mg L^−1^, and T_3_ 20.80 mg L^−1^, and a control 0 mg L^−1^ following the 96 h LC_50_ of 51.96 mg L^−1^ from acute toxicity test. Overall growth performance significantly declined in all the Pb(NO_3_)_2_ treated groups and the highest mortality was recorded in T_3_. Behavioural abnormalities were intense in all the treatment groups compared to the control. Hepatosomatic index (HSI) values were reported as higher in treatment groups. Reduced nucleus diameter and nuclei size in erythrocytes were reported for T_2_ and T_3_ groups. Dose-dependent histological alterations were visible in the gills, liver, and intestine of all the Pb(NO_3_)_2_ treated groups. The width of the intestinal villi was highly extended in T_3_ showing signs of severe histological alterations. In conclusion, Pb toxicity causes a negative effect on growth performance, erythrocyte morphology, and affected the vital organs histomorphology of juvenile *O. niloticus.*

## 1. Introduction

Tilapia is one of the most significant commercial species which has emerged as aquaculture’s key species around the world [1,2]. The Nile tilapia, *Oreochromis niloticus*, alone contributes almost 80% of total farmed tilapia production globally [3]. Tilapia is a tenacious, fast-growing farmed fish, considered to have great potential, especially in low-income nations. It has been revealed to play key roles in financial and nutritional support for the rural poorest people [4,5,6]. It has an outstanding capacity to survive in a wide thermal range and adverse environmental conditions, which exhibits them as a potential bio-indicator of aquatic pollution [7,8]. Henceforth, tilapia in recent times has become a model fish to study toxicity in a particular aquatic habitat [9,10].

Almost all industrial toxic effluents, as well as anthropogenic outflows, eventually end up in aquatic ecosystems [11,12]. Because of the rapid economic development across the globe, large-scale emissions and pollution by heavy metals are of special concern. The frequent discharges of those exaggerated pollutants into water bodies cause harmful effects on aquatic living creatures. Heavy metals disrupt the ecological equilibrium by interfering with a variety of physiological, metabolic, and cellular functions of living organisms [13,14]. At higher concentrations, they become a concern for aquatic habitats as often the biological system itself alone is unable to destroy those kinds of substances rapidly [15,16]. Excess quantity of heavy metals in water produces Reactive Oxygen Species (ROS), which collapse the water quality and poses a high risk for aquatic life by causing oxidative stress [15,17,18]. Lead (Pb), is one of the momentous toxins and persistent heavy metals in aquatic ecosystems, which is responsible for the deterioration of ecological health in aquatic environments as well as a big threat to living creatures [15,17]. Chronic exposure to the Pb toxicant has been reported to intensify the production of reactive oxygen species (ROS), causing oxidative damage and abnormal proliferation of free radicals [19,20]. Pb is bioaccumulated in aquatic systems through the water and food, or via inactive absorption by the fish gills or skin. Afterward, it has the immense potential to accumulate in vital fish organs, for instance, the liver, gills, kidney, and digestive tract [21,22]. Lead poisoning causes disturbance in fish physiology, damages blood cells, and brings significant alterations in body tissues [16,17]. Higher Pb accumulation in water bodies could lead to higher permeation of Pb in the fish body, which ultimately indicates the potentiality of causing deleterious effects on consumers’ health [17,23].

To study the toxicity in fish, nowadays, advanced and standardised acute toxicity tests are being applied, which are quite useful in environmental risk assessment [24,25]. Lethal concentration (LC_50_), for instance, is globally deemed an effective tool to study environmental toxicology [26,27]. Furthermore, the status of toxicants can also be assessed through the study of haematology and erythrocyte morphology [15,28]. Heavy metal toxicity in fish has conventionally been investigated through histopathological examinations [29,30]. Histological investigation of vital fish organs, toxicity assays, and growth metrics are thought to be powerful tools to study heavy metal toxicity in fish [31,32]. Therefore, the current study aimed at approaching multiple biomarkers, i.e., growth, behavioural anomalies, blood cell morphology, and tissue structure of vital organs in juvenile Nile tilapia, *O. niloticus*, to assess chronic Pb toxicity.

## 2. Materials and Methods

### 2.1. Collection and Acclimatization of Fish

**Figure 1:**
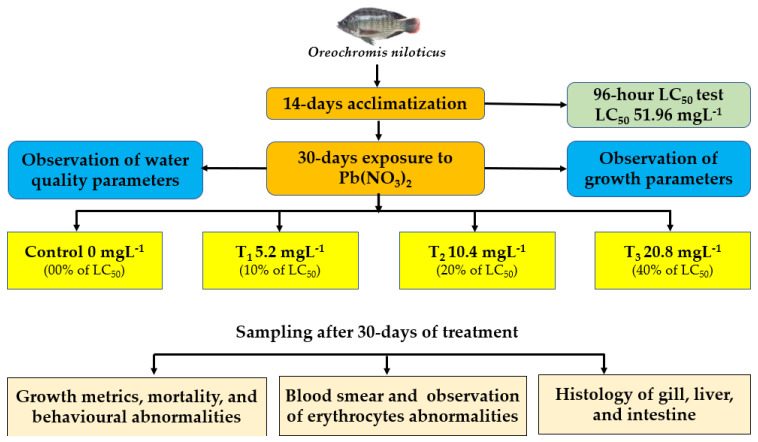
A flow chart illustrating the overall steps of the methodology.

The study was conducted in the wet laboratory of the Department of Fish Biology and Genetics, Sylhet Agricultural University, Sylhet. About 500 fries of Nile tilapia, *O. niloticus* were obtained in February 2022 from a commercial hatchery of Sreemangal and transported to the Sylhet Agricultural University in aerated polythene bags. The fish were allowed to acclimatise in large, aerated plastic drums for 2 weeks. At this time, the fish were fed twice a day at a rate of 5% of their body weight. Figure 1 demonstrates the overall methodology and assays conducted in the current research.

### 2.2. Acute Toxicity Test, Experimental Designing, and Feeding

A 96 h lethal concentration (LC_50_) test for tilapia fish was conducted by using thirteen doses of Pb(NO_3_)_2_ (0, 10, 20, 30, 40, 50, 60, 70, 80, 90, 100, 120, and 130 mg L^−1^). Thirteen separate glass aquaria were loaded with 60 L dechlorinated tape water, where 20 fries were randomly stocked in each and continuously aerated with centrally installed air pumps. The mortality rate was recorded at each 12 h interval. The resulting mortality rate was transformed into a Probit value, thereafter, the LC_50_ value was computed by using linear regression between Probit variables and the logarithmic value of toxicants concentration according to the standard procedures of Finney [33].

Afterward, twelve glass aquariums were used for the next experimental step, where the length, width, and depth of each aquarium were 73.5 cm, 35.5 cm, and 38.0 cm, respectively. At the very beginning, all the aquaria were cleaned properly with dechlorinated water, henceforth, filled with 60 L of clean tap water, and provided suitable aeration through a 2 hp air-blower system. Afterward, 20 uniform-sized fish were randomly distributed in each aquarium. Ambient daylight and the dark regimes were constant in the tanks. Water was swapped at a frequency of 20% of tank volume two times per week. Debris, unused feed, and faecal contents were siphoned twice a day to ensure an adequate water environment for fish existence. To evaluate fish populations and readjust the nutrition, quarterly surveys were carried out using a scope net. The tilapia’s activity had also been constantly monitored, specifically after feeding. Then animals were assigned to four treatment groups (Table 1) following the LC_50_ value from the toxicity test and reared for 30 days.

A starter diet (consisting of fishmeal 16%, peanut meal 24%, soybean meal 14%, rice bran 30%, broken rice 15%, and vitamin/mineral premixes 1%) was used to feed the fish (Aftab Feed Product Ltd., Dhaka, Bangladesh). Fish were fed twice a day at 12 h intervals (10:00 a.m., and 10:00 p.m., powdered diets supplement). The feeding rate was consistent among treatments, extending from 4 to 6% of body weight. Following biweekly sampling, the feeding rate was tweaked. Residual feed and excrement were retrieved from the tanks regularly, and any mortality was documented.

### 2.3. Acquisition of Physicochemical Properties of Rearing Water

The water quality parameters such as temperature, dissolved oxygen (DO), pH, and salinity were monitored three times (1st, 15th, and 30th days) by using a professional YSI digital multi-Probe meter, Model 58. A commercial ammonia kit manufactured by HACH (Model NI-SA, India) was used to measure the ammonia.

### 2.4. Analysis of Behavioural Abnormalities

Different behavioural abnormalities were noted regularly and expressed in a semi-quantitative approach by using the methodology of Misra and Mohanty [34] and Hossain et al. [35].

### 2.5. Tools for Growth Metrics

The following equations were used to compute the specific growth rate (SGR), condition factor (*K*), and hepatosomatic index (HSI).

Specific growth rate (SGR) in % /day = $\frac{ln\left(\;wt\right)-ln\;\left(\;w0\right)}{t}\times 100$. Here, *wt* refers to the final weight and *w*0 initial body weight of the individual, respectively, and t is the duration of the trial in days.

Fulton’s condition factor, *K* = WL^−3^ × 100 [36]. Here, W is the weight (g) and L is the length (cm) of an individual fish (g), [37]

$$\mathrm{Hepatosomatic}\;\mathrm{index}\;(\mathrm{HSI})\;=\;\frac{Liver\;weight}{Body\;weight}\times 100$$

### 2.6. Sampling and Bleeding Fish

Weight and length measurements were performed on the 15th and 30th days. An electronic weighing machine (CAMRY digital electrical balance, Model EK 3052, Bangladesh) was used to measure the weight, and a centimetre scale was used to estimate the length. At the end of the experimental periods, blood was collected from the caudal vein of five fish from each replicate of all treatment groups to prepare a blood smear from a freshly collected sample. Five fish from each replication of four treatments were slaughtered to collect the gills, liver, and intestine for histology examination, and tissue samples were immediately fixed in 10% neutral buffered formalin.

### 2.7. Preparation of Blood Smear and Histology of Gill, Liver, and Intestine

Thin smears on pre-cleaned slides for all collected blood samples were prepared to perform erythrocyte analysis. After that, the slides were air-dried and fixed into methanol solution for 5 min before counterstaining in Giemsa stain for 10 min. Then the slides were washed with running tap water and air-dried overnight. Prefixed samples in neutral buffered formalin went through the standard histological procedure described by Slaoui and Fiette [38]. Gill, liver, and intestine cell sections were visualised at different magnifications by using a light microscope (Primo Star, ZEISS, Jena, Germany) equipped with a camera (Axiocam, ZEISS, Jena, Germany) and run-on ZEN core version 3.0 Windows software. About ten slides from each organ have been examined and resulted pathologies were recorded for quantitative analysis. Histopathology was identified by following the previous literature [39,40]. The pathology noted below 5% in total observation has been referred to as absent (---), 6–25% as weak (*), 26–50% as moderate (**), and above 50 % as severe (***) as per as the description of Ekpenyong et al. [41].

### 2.8. Statistical Analysis

All raw data were processed in Microsoft Excel, and afterward, all analyses were performed by using SPSS v26. Means were compared in ANOVA, and Tukey’s HSD post-hoc test was used to determine the significant differences between treatments at *p* < 0.05.

## 3. Results

### 3.1. Acute Toxicity Test

**Figure 2:**
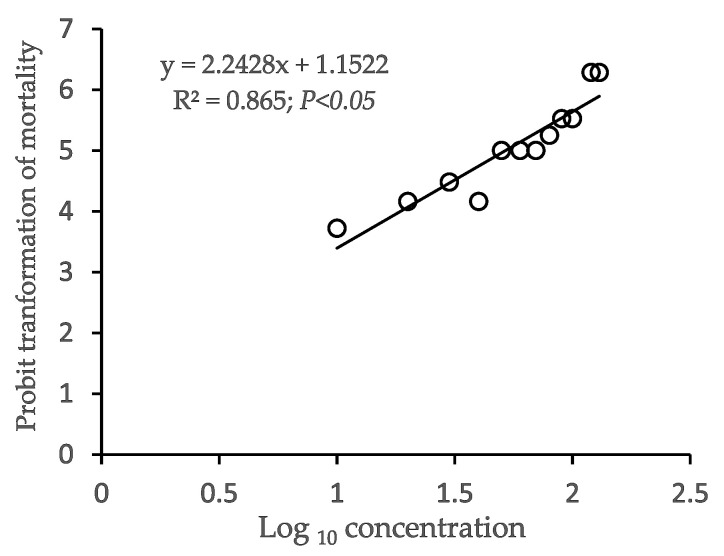
The 96 h Pb(NO_3_)_2_ LC_50_ regression curve for *O. niloticus*.

The 96 h LC_50_ of Pb(NO_3_)_2_ for *O. niloticus* was computed as 51.96 mg L^−1^ in the present study. Figure 2 depicts the regression between the logarithmic concentration of Pb(NO_3_)_2_ and the Probit transformation of mortality in *O. niloticus*. The susceptibility of individual lethality was enhanced with an increase in lead concentration, whereas mortality was essentially non-existent in the control. Therefore, the trial remained within the standard conditions of OECD guidelines for acute toxicity tests [25]. Current values of LC_50_ have been justified by previous acute toxicity tests in different tilapia species of the *Oreochromis* genus (Table 2).

### 3.2. Water Quality Assessment

Table 3 elucidates the physicochemical features of water during the trial period. On the one hand, salinity, pH, and NH_3_ concentration remained almost stable in all treatment groups during the experiment. DO level, on the other hand, declined from 8.14 ± 0.08 to 6.64 ± 0.15 mg L^−1^ among the different time slots with a slight increase of water temperature from 19.47 ± 0.06 to 20.30 ± 0.26 °C.

### 3.3. Growth Performance, and Hepatosomatic Index (HSI)

**Figure 3:**
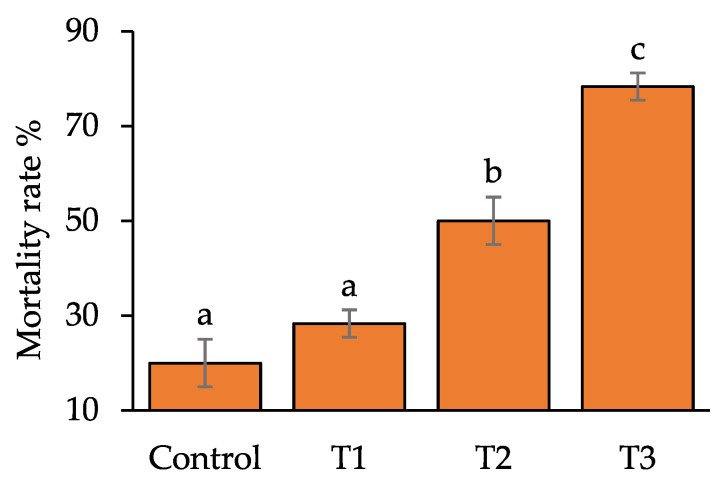
The mortality rate of O. niloticus in different treatment groups. Different superscripts indicate significant differences at *p* < 0.05.

**Figure 4:**
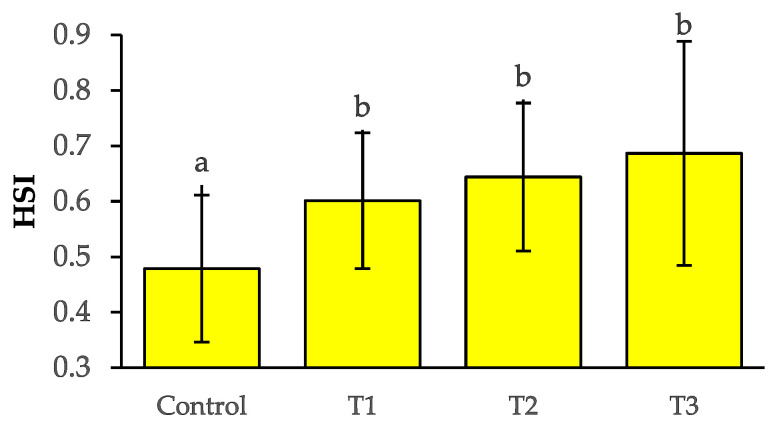
Hepatosomatic (HSI) indices in different treatment groups. Different superscripts indicate significant differences at *p* < 0.05.

The growth performance of *O. niloticus* is summarised in Table 4. It is reported that final length and weight, condition factor (K), specific growth rate (SGR), and length and weight gain percentages were significantly (*p* < 0.05) decreased in groups exposed to Pb(NO_3_)_2_ in comparison to the control group. The statistically highest (*p* < 0.05) final length and weight were observed in the control group as 4.40 ± 0.09 cm and 1.47 ± 0.08 g, respectively, while the lowest values were documented in T_3_ as 3.55 ± 0.03 cm and 0.52 ± 0.03 g, respectively, followed by T_2_ and T_1_. The value of Fulton’s condition factors, K, also followed a similar trend. The highest SGR % was 2.39 ± 0.20 in the control group; on the contrary, the lowest was −1.03 ± 0.24 in T_3_ followed by T_2_ and T_1_. The highest mortality was noted in T_3_ followed by T_2_, while both were statistically higher than the control (*p* < 0.05) (Figure 3). HSI values were increased for all the Pb(NO_3_)_2_ treated groups (Figure 4).

### 3.4. Behavioural Abnormalities

No behavioural abnormalities were noted for the control group (Table 5). However, the significant onset of behavioural alterations associated with feeding, movement, and coloration in skin and gills were noted as prominent in all treatment groups (Table 5). Most of the behavioural abnormalities were very intense in T_2_ and T_3_ treatments at the end of 30 days of the trial period.

### 3.5. Erythrocyte Abnormalities

**Figure 5:**
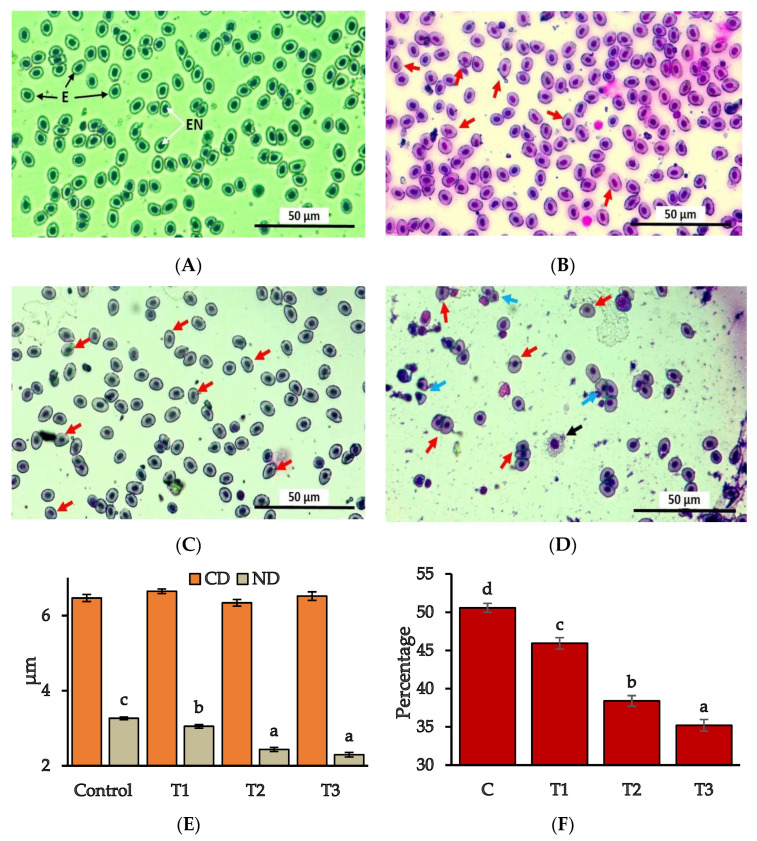
Microscopic view of erythrocytes in different treatment groups; (**A**) Control, (**B**) T_1_, (**C**) T_2_, (**D**) T_3_. (E—erythrocytes, EN—elliptical nuclei; red arrows—shrinking nuclei, black arrows—erythrocytes with rupturing cell membrane, blue arrows—shape deformities); (**E**,**F**) Quantitative analysis of erythrocytes in different treatment groups. Different superscripts indicate significant differences at *p* < 0.05 (CD—cell diameter, ND–nucleus diameter).

Erythrocyte abnormalities were identified by following the description of Shahjahan et al. [45] and Sayed et al. [46]. Normal erythrocytes with elliptical nuclei were spotted on the peripheral blood of control individuals (Figure 5A). Erythrocyte density gradually declined with the enhancement of Pb(NO_3_)_2_ concentrations (Figure 5B–D). Moreover, a few erythrocyte abnormalities were identified in the exposed groups (Figure 4). On the other hand, Figure 5E shows a significant difference in terms of nucleus diameter (ND) between the control and exposed groups. There was no statistical difference in cell diameter (CD) among the four treatment groups, while the nucleus diameter remained highest for the control group and significantly reduced in all the treatment groups (*p* < 0.05). Figure 5F illustrates the percentages of nuclei in erythrocytes in all treatment groups, which refers to the quantitative indication of nuclear deformation in treatment groups in comparison with the control (*p* < 0.05).

### 3.6. Histopathology in Gills, Liver, and Intestine

**Figure 6:**
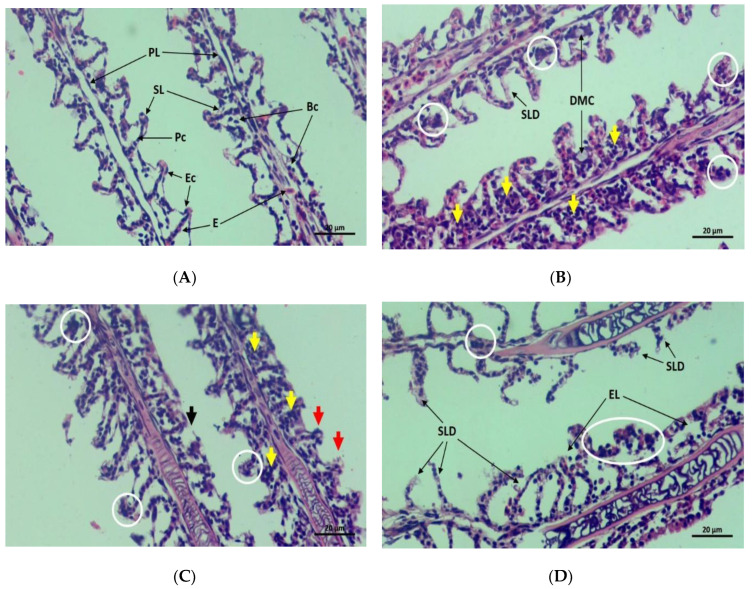

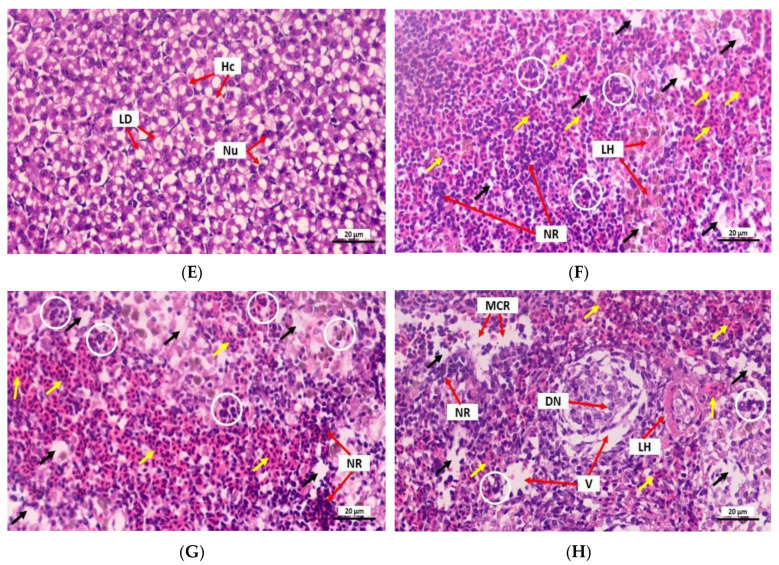
Longitudinal microscopic view of gills. (**A**) Control, (**B**) T_1_, (**C**) T_2_, (**D**) T_3_ (PL—primary lamellae, SL—secondary lamellae, Pc–pillar cells, Ec—epithelial cells, E—erythrocytes, Bc—basal cells, DMC–diffusion of mucous cells, SLD—secondary lamellae damage, EL—epithelial lifting; white circle—acute necrosis, yellow arrows—congestion of basal cells, red arrows—shortening secondary lamellae, black arrows—damage of epithelial layer). Transverse photomicrographs of liver. (**E**) Control, (**F**) T_1_, (**G**) T_2_, (**H**) T_3_ (Hc—hepatocytes, Nu—nuclei, LD—lipid droplets, LH–liver haemorrhage, NR–nuclear ruptures, DN—degenerated nuclei, MCR—massive cell rupture, V—vacuole; white circle—necrosis, black arrows—cell rupture, yellow arrows—erythrocyte infiltration in blood sinusoids).

**Figure 7:**
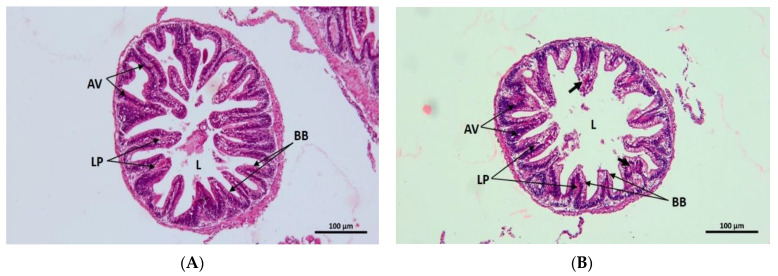

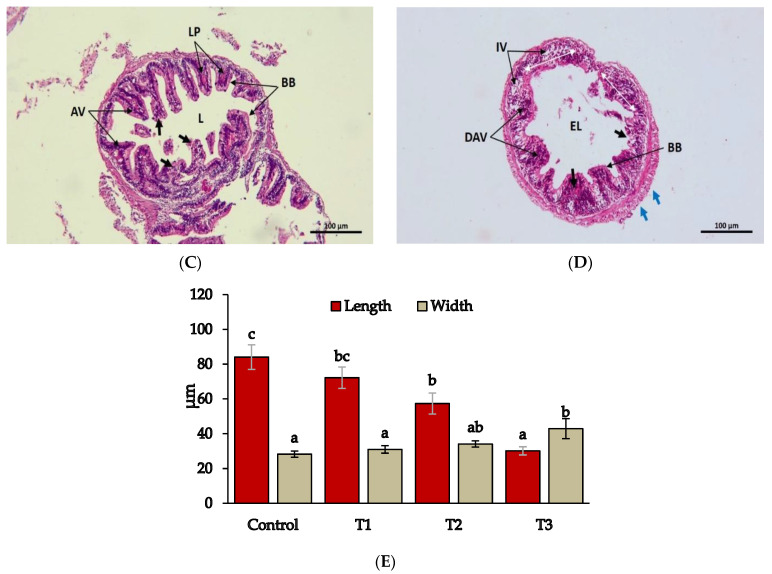
Transverse photomicrographs of the intestine. (**A**) Control, (**B**) T_1_, (**C**) T_2_, (**D**) T_3_. (BB—brush border, AV—absorptive vacuole, LP—lamina propria, L—lumen, EL—extended lumen, IV—increased vacuoles, DAV—disarranged absorptive vacuole; black arrows—tissue rapture, blue arrows—extended serosa, white both side arrows—wider villi); (**E**) Length and width of intestinal villi in different treatment groups. Different superscripts indicate significant differences at *p* < 0.05.

Gills in control groups refer to a healthy condition with well-structured primary and secondary lamellae, pillar cells, epithelial cells, erythrocytes, and basal cells (Figure 6A). On the contrary, gills from treatment groups were affected mostly by secondary lamellae damage, acute necrosis, and congestion of basal cells (Figure 6B–D). Apart from those abnormalities, diffusion of mucous cells also appeared in T_1_ (Figure 6B). Shortening secondary lamellae and damage to the epithelial layer were noticed in fish gills from T_2_ (Figure 6C), and epithelial lifting was also reported in T_3_ (Figure 6D). Healthy liver tissues with normal hexagonal hepatocytes, prominent nuclei, and abundant lipid droplets were noted in the control group (Figure 6E). Several abnormalities were accounted for in treatment groups, for instance, liver haemorrhage, nuclear ruptures, necrosis, cell rupture, and erythrocyte infiltration in blood sinusoids (Figure 6F–H). The T_1_ and T_2_ were reported to be highly affected by necrosis, cell ruptures, erythrocyte infiltration, and nuclear ruptures (Figure 6F,G). Again, degenerated nuclei, massive cell ruptures, as well as large vacuoles due to cell ruptures were recorded in the hepatic tissues from T_3_ (Figure 6H). A firmed intestinal wall and villi containing brush borders, absorptive vacuoles, lamina propria, and lumen in the centre were in the control group (Figure 7A), while tissue ruptures were marked in treatment groups (Figure 7B–D). The T_3_ was predominantly affected by an extended lumen, increased vacuoles, disarranged absorptive vacuoles, extended serosa, and wider villi (Figure 7C).

The length of intestinal villi gradually decreased when compared with the control and the lowest value was obtained in T_3_ (*p* < 0.05) (Figure 7E). In terms of the width of intestinal villi, the highest measurement was recorded in T_3_, indicating the severest form of disruption among the treatment groups. A comparative investigation of the current histopathological analysis of the gills, liver, and intestine has been organised in Table 6.

## 4. Discussion

The LC_50_ is a widely used tool in toxicity research of aquatic animals. The current value of 96 h LC_50_ Pb(NO_3_)_2_ is 51.96 mg L^−1^ for *O. niloticus*, which seemed aligned with the previous investigations where it was noted between 40 to 44 mg L^−1^ [17,44]. In contrast, lower values of 11.05 mg L^−1^ [18], 17.33 mg L^−1^ [42], and 18.70 mg L^−1^ [43] have been documented for the same species. These wide variations denoted that the sensitivity to Pb differs between species, age, and size, and depends on chemical formulations of toxicants [47,48], and physicochemical characteristics of the experimental environments [9,49]. Major physicochemical features of water, i.e., temperature, DO level, and pH were maintained as essentially optimal over the exposure time following the standard requirements of Chapman et al. [50].

Fish show behavioural alterations and unusual movements due to physiological and metabolic disturbances caused by exposure to toxicants [9,47]. Accounts of maximum mortality in the highest treatment groups, T_3_ in current research agreed with Brraich et al. [48], while the mortality rate was increased with the high concentration dosages. Increased mortality was also noted in *C. punctatus* and *H. fossilis* when exposed to Pb(NO_3_)_2_ [51]. It had been well-studied that heavy metal exposure causes a detrimental impact on the growth performance of *O. niloticus* [52]. The addition of 0.075 mg L^−1^ Pb for 60 days was reported to significantly reduce the size and weight of the fry [53]. Lower growth performance in the current trial was similar to previous studies which revealed reduced growth of fishes exposed to toxicants or other means of pollutants [9,17,52]. Poor growth performance in the treatment groups in comparison to control groups is due to less utilization of food, and weak physiological conditions associated with compensation of toxicity stress [54,55]. The poisonous effect of different toxic content in fish can be chased by quantifying behavioural abnormalities [35,56]. Erratic locomotion and gasping for air were notable behavioural signs in *Labeo rohita* exposed to arsenic [57]. The behavioural alterations in current research remained aligned with the investigation of Ekpenyong et al. [41] and Okor et al. [56]. Elevated HSI was observed in *O. niloticus* exposed to chlorpyrifos pesticides [55]. Heavy metals were also responsible for raising the HSI values by initiating hepatic tissue abnormalities and elevating the breakdown of liver enzymes [58]. Increased HSI was reported by the current study, which might be a result of gaining extra mass due to liquid congestion in the vacuoles at higher exposure levels, which was also identified by Hossain et al. [9] for the same species exposed to organophosphate toxicants.

A number of erythrocyte abnormalities were noted in several studies with fish in response to diverse toxicants; for instance, an increase of lymphocytes proliferation in *O. niloticus* [59], morphological changes of shape-shifted red blood cells in *O. mykiss* [60], and *O. niloticus* [45], erythrocytic nuclear alteration in tilapia [61], and micronuclei induction in *Channa punctatus*, [30] and *Pangasianododon hypophthalmus* [62]. The diameter of erythrocytes and nucleus were reported to be affected by the environmental parameters of the living medium [63]. Above mentioned deformities were common in different treatment groups of the present study. Again, shape deformities, cell ruptures along with the lower nuclear diameter, and decreasing the area of nuclei in erythrocytes are associated with the breakdown of the cytoskeleton due to toxicant stress [45,64].

Histological analysis of vital fish organs is a significant method to study the severity of heavy metal toxicity which has already been performed by several authors [65,66,67]. Gill and liver histology in fish are considered notable biomarkers in environmental toxicology research [15,68]. Gills are very sensitive to any kind of toxicants due to their extended surface and continuous uptake of water from the surrounding environment [69]. This unique characteristic represents it as an excellent bio-indicator of aquatic pollution [15]. Hossain et al., [9] recorded the shortening of gill lamellae, damage of filament, necrosis in epithelial tissue, epithelial lifting, and mucous cell diffusion were major pathological signs in *O. niloticus* gills exposed to chlorpyrifos. Significant alterations were visible in the primary and secondary gill lamellae of *C. carpio* exposed to Pb(NO_3_)_2_ [15]. Barbieri [67] also noted similar pathologies in the case of Pb toxicity in tilapia. The above results are agreed with the current findings. Kiran et al. [17] reported that the inflammation, mild and severe haemorrhage, necrosis, vacuolation, and dilation of hepatic sinusoids were prominent pathologies in *O. niloticus* exposed to the same toxicant. Fish exposed to different levels of stress and toxicant revealed intensive hepatic haemorrhages, necrosis, and vacuolation [14,70]. Mild haemorrhage, high lipid content, and loss of sinusoidal area were observed in Cu-exposed *O. niloticus*, while increased vacuoles, degenerated nuclei, erythrocyte infiltration, and haemorrhage were the major findings from Cd-treated *O. niloticus* [71]. Very similar histological changes were noted in the current research. The intestine is one of the vital organs for digestion and is major for nutrient absorption in fish [72]. Degeneration and necrosis in the intestinal mucosa, oedema, and atrophy in submucosa and muscularis were common detections in the intestinal tissue of *O. niloticus* exposed to Cd [73]. The higher concentration of Pb(NO_3_)_2_ in the current research also showed significant changes in the intestine including damage to villi, increased evacuation, and shortening of villi height. Therefore, histological evidence from the current investigation suggested that the experimental exposure to Pb(NO_3_)_2_ induced robust histomorphological alterations in gill, intestinal, and hepatic tissue of Nile tilapia.

## 5. Conclusions

The findings of current research suggested that Pb pollution in water posed a remarkable reduction in growth performance due to toxicant-born stress and related physiological compensation in *O. niloticus.* Additionally, a higher rate of mortality in high-dose treatment units endures the Pb-induced lethality in the studied species. It has also been noted that Pb pollution brings significant histomorphological alterations in blood cells and vital organs tissue structure. 

## Figures and Tables

**Table 1:** Experimental design with different dosages of Pb(NO_3_)_2_ exposure.

Treatment	Concentration of Pb(NO_3_)_2_ (mg L^−1^)	Stocking Density (No./Replicate)	Replication
Control	00.00 (00% of LC_50_)	20	3
T_1_	05.20 (10% of LC_50_)	20	3
T_2_	10.40 (20% of LC_50_)	20	3
T_3_	20.80 (40% of LC_50_)	20	3

**Table 2:** The 96 h LC_50_ values for different formulations of Pb in the *Oreochromis* genus.

Species	Value of 96-h LC_50_ (mg L^−1^)	Formulation of Toxicant	References
*O. niloticus*	51.96	Pb(NO_3_)_2_	Current study
*O. niloticus*	40.29		[17]
*O. mossambicus*	17.33	Pb(C_2_H_3_O_2_)_2_	[42]
*O. mossambicus*	18.70		[43]
*O. niloticus*	44.0	Pb(NO_3_)_2_	[44]
*Oreochromis* sp.	11.05		[18]

**Table 3:** Physicochemical properties of water in different treatment groups.

Parameters	Treatment	Day 0	Day 15	Day 30
Temperature (°C)	Control	19.93 ± 0.12	20.06 ± 0.04	20.30 ± 0.21
	T_1_	19.80 ± 0.10	19.90 ± 0.06	20.20 ± 0.03
	T_2_	19.47 ± 0.03	10.87 ± 0.07	20.13± 0.08
	T_3_	19.53 ± 0.07	19.97 ± 0.13	20.20 ± 0.08
pH	Control	8.27 ± 0.06	8.16 ± 0.05	8.06 ± 0.03
	T_1_	8.23 ± 0.07	8.19 ± 0.07	8.13 ± 0.03
	T_2_	8.23 ± 0.04	8.2 ± 0.06	8.17 ± 0.03
	T_3_	8.21 ± 0.07	8.14 ± 0.06	8.13 ± 0.07
Salinity	Control	0.07 ± 00	0.08 ± 0.01	0.10 ± 00
	T_1_	0.07 ± 0.01	0.07 ± 0.01	0.09 ± 0.02
	T_2_	0.06 ± 0.01	0.08 ± 0.02	0.10 ± 0.01
	T_3_	0.07 ± 0.03	0.08 ± 0.01	0.09 ± 0.01
NH_3_ (mg L^−1^)	Control	0.02 ± 0.01	0.02 ± 0.01	0.03 ± 0.01
	T_1_	0.01 ± 0.02	0.03 ± 0.01	0.03 ± 0.01
	T_2_	0.02 ± 0.01	0.04 ± 0.01	0.03 ± 0.01
	T_3_	0.02 ± 0.00	0.03 ± 0.01	0.03 ± 0.01
DO (mg L^−1^)	Control	7.77 ± 0.10	7.06 ± 0.17	6.96 ± 0.04
	T_1_	8.11 ± 0.07	7.13 ± 0.06	6.96 ± 0.05
	T_2_	8.05 ± 0.16	6.79 ± 0.02	6.64 ± 0.09
	T_3_	8.14 ± 0.08	6.57 ± 0.08	6.73 ± 0.08

The column with different superscripts indicates significant differences at *p* < 0.05; values are means ± SE.

**Table 4:** Growth performance of *O. niloticus* exposed to Pb(NO_3_)_2_ at different concentrations.

Parameters	Control	T_1_	T_2_	T_3_
Initial length (cm)	3.44 ± 0.05	3.43 ± 0.06	3.43 ± 0.08	3.45 ± 0.03
Initial weight (g)	0.70 ± 0.01	0.69 ± 0.03	0.69 ± 0.04	0.70 ± 0.02
Final length (cm)	4.40 ± 0.09 ^c^	3.82 ± 0.05 ^b^	3.70 ± 0.07 ^ab^	3.55 ± 0.03 ^a^
Final weight (g)	1.47 ± 0.08 ^c^	0.78 ± 0.05 ^b^	0.55 ± 0.04 ^a^	0.52 ± 0.03 ^a^
K	1.7 ± 0.04 ^c^	1.36 ± 0.05 ^b^	1.10 ± 0.07 ^a^	1.16 ± 0.06 ^a^
SGR %	2.39 ± 0.20 ^c^	0.32 ± 0.26 ^b^	−0.77 ± 0.34 ^a^	−1.03 ± 0.24 ^a^
Length gain %	28.35 ± 3.15 ^c^	12.08 ± 2.48 ^b^	8.23 ± 2.13 ^ab^	3.18 ± 0.80 ^a^
Weight gain %	110.40 ± 11.39 ^c^	15.94 ± 8.94 ^b^	−13.28 ± 8.72 ^a^	−23.60 ± 4.82 ^a^

The column with different superscripts indicates significant differences at *p* < 0.05; values are means ± Standard Error.

**Table 5:** Behavioural abnormalities of fishes among the different treatment units.

Treatment	Day	Abnormalities					
		Loss of Appetite	Gasping for Air	Sluggish Movement	Erratic Locomotion	Pale Gills	Skin Color Change
Control	15	--	--	--	--	--	--
T_1_		--	--	*	--	--	--
T_2_		*	*	*	--	*	*
T_3_		*	*	**	*	*	*
Control	30	--	--	--	--	--	--
T_1_		*	*	**	*	**	**
T_2_		**	*	**	**	*	**
T_3_		**	**	***	**	**	***

-- normal, * weak (<10%), ** moderate (10–50%), and *** severe (>50%).

**Table 6:** Comparative investigation of histopathological alterations from the current experiment in different treatment units.

Organ	Abnormality	Control	T_1_	T_2_	T_3_
Gill	Diffusion of mucous cells	---	*	---	---
	Secondary lamellae damage	---	*	*	***
	Epithelial lifting	---	---	---	**
	Acute necrosis	---	**	***	***
	Congestion of basal cells	---	***	**	---
	Shortening secondary lamellae	---	---	**	---
	Damage to the epithelial layer	---	---	**	**
Liver	Liver haemorrhage	---	***	***	**
	Nuclear ruptures	---	***	***	**
	Degenerated nuclei	---	---	---	**
	Massive cell rupture	---	---	---	***
	Vacuole caused by cell rupture	---	---	---	**
	Necrosis	---	**	***	**
	Cell ruptures	---	**	***	***
	Erythrocyte infiltration in blood sinusoids	---	**	***	**
Intestine	Extended lumen	---	---	---	**
	Increased vacuoles	---	---	---	***
	Disarranged absorptive vacuoles	---	---	*	***
	Tissue rapture	---	*	*	*
	Extended serosa	---	---	---	*
	Wider villi	---	*	*	***

Histopathological alterations are referred to as absent (---), weak (*), moderate (**), and severe (***).

## Data Availability

All the raw and analyzed data will be available from the corresponding author based on reasonable demand.
